# Impact of Stoma Baseplate Convexity on Tension and Compression Around the Stoma Site: A Finite Element Analysis

**DOI:** 10.7759/cureus.52112

**Published:** 2024-01-11

**Authors:** Jonathan Waller, Philip Gowans, Suzanne Lord, Katie McGill

**Affiliations:** 1 Engineering, Kinneir Dufort Design Ltd., Bristol, GBR; 2 Ostomy Care, Convatec Ltd., Deeside, GBR; 3 Ostomy Care, Convatec Ltd., Lexington, USA

**Keywords:** wound care, ostomy care, urinary stomas, intestinal stomas, compression, tension, convexity, stoma, finite element

## Abstract

For patients living with intestinal or urinary stomas, skin barriers play an essential role in protecting the peristomal skin and preventing peristomal complications. Convex baseplates press into the peristomal skin and are suitable for retracted stomas that do not protrude, peristomal skin with creases, folds, or dips, and stomas where frequent leaking can occur with flat pouching systems. However, there is a lack of data on the magnitude and location of tension applied to the abdomen by convex baseplates. We evaluated the impact of a range of convex baseplates applied to a simulated stoma site. A comparative finite element analysis investigation was conducted to evaluate the impact of eight different convex stoma system baseplates applied to an idealised flat abdomen, representing skin, subcutaneous tissue, and musculature layers. The baseplates considered had varying convexity with depths of 3.5 mm and 7 mm and internal structural diameters between ~30 mm and ~60 mm. The convex product range provided tension in the skin (maximum principal strain) and compression through the fat layer (minimum principal strain). Large differences in the locations and magnitudes of skin tension and fat layer compression were seen between the baseplates under analysis, with both the depth and diameter of convexity influencing the strain experienced across the abdominal topography. The results generated highlight the importance of having an appropriate range of convexity products available and selecting an appropriate option for use based on the stoma type and condition of the peristomal skin.

## Introduction

Approximately one million people in the United States and 750,000 people in the European Union are estimated to be living with a stoma [1,2]. In ostomy care, a primary goal is establishing a secure and predictable seal between the peristomal skin and the baseplate of the stoma system [3]. Peristomal skin complications as a result of leaked effluent (e.g., peristomal moisture-associated skin damage) is common among people living with stomas and there are a number of solutions to improve this seal, from pastes and separate sealing components to convex baseplate designs that press into the peristomal skin [4,5]. While a flat baseplate design is typically suitable for patients with a budded stoma and flat peristomal skin, the curvature of a convex baseplate can better suit flush and retracted stomas that do not protrude, or peristomal skin with creases or folds [3,6]. For individuals who require convexity, a key challenge is selecting a correct fitting baseplate to maintain an adequate seal while minimising the risk of pressure injuries [3,5,6]. This challenge is compounded by significant patient-to-patient variation such as differences in skin folds, levels of abdominal fat, skin fragility, stoma size, and stoma protrusion [7].

There is a range of commercially available convex products with different characteristics to suit individual patient needs. An international consensus panel defined five fundamental convex product characteristics: depth, compressibility, flexibility, slope, and tension location [6]. Selecting appropriate values for these characteristics can allow a good seal to be generated while avoiding excess pressure on the peristomal skin [6]. However, there is a lack of data on the magnitude and location of tension applied on the abdomen by convex baseplates, limiting their effective and consistent implementation in clinical practice. To evaluate the effect of baseplate convexity on a stoma site with regard to skin tension and fat compression, we conducted a finite element (FE) analysis simulating the application of convex baseplates with different geometries and flexibilities to an abdomen, and compared the relative reactions in the abdomen.

## Technical report

Methods

Stoma System Baseplates

**Figure 1:**
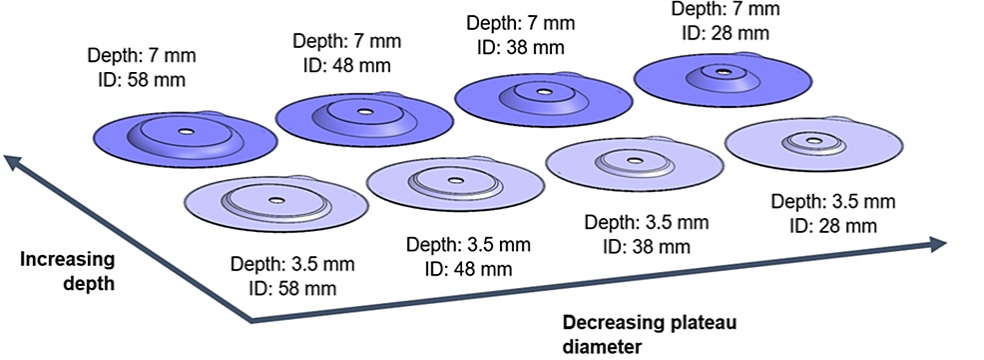
Range of stoma system baseplates ID: inner diameter.

**Figure 2:**
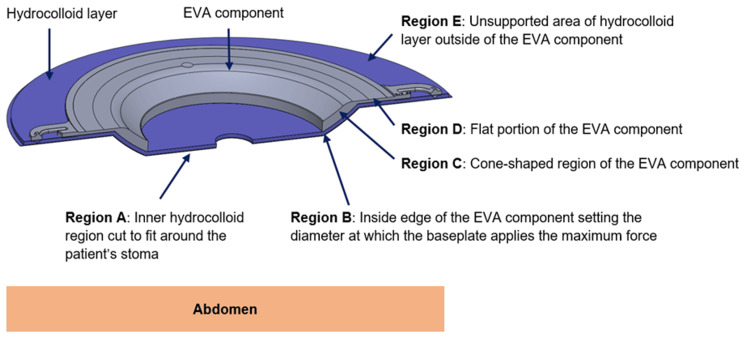
Key regions of stoma system baseplates Cross-sectioned view of the 7 mm depth–38 mm diameter baseplate. EVA: ethylene-vinyl acetate.

Eight stoma system baseplates (Convatec Ltd., Deeside, United Kingdom) with a range of inner diameters (28-58 mm) and depths (3.5 mm or 7 mm) were compared in this analysis (Figure 1). The baseplates are comprised of a flexible hydrocolloid layer that adheres to the skin and an injection moulded ethylene-vinyl acetate (EVA) component that provides structural support (Figure 2). The hydrocolloid layer has an inner region (A) that is cut to fit around the stoma and an unsupported outer region (E). The EVA component has an inside edge (B) that pushes into the abdomen and applies force to the peristomal skin, a cone-shaped region (C), and a flat region (D). The inner diameters of the baseplates were designed to apply force at different locations, with smaller inner diameters influencing the skin nearest the stoma (e.g., stomas that do not protrude above the skin) and larger inner diameters helping to smooth out and stabilise skin away from the stoma (e.g., skin with folds or creases).

FE Analysis Model Setup

**Figure 3:**
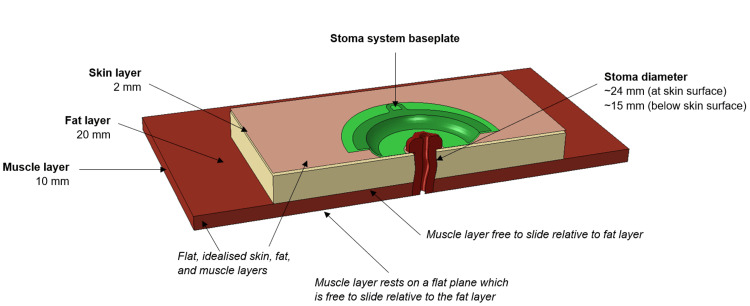
Abdomen model with idealised (flat) geometry and stoma baseplate for analysis Information gathered from the literature review defined the parameters for skin [8-10], fat [8,10-12], and muscle [12,13] layers.

**Figure 4:**
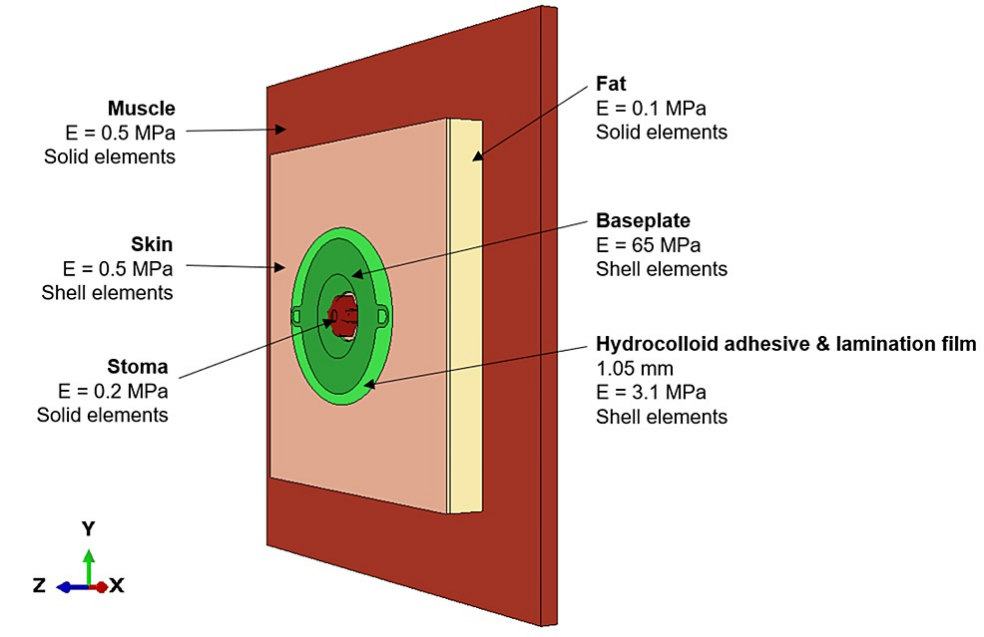
Material properties applied to each region of the flat abdomen model Information gathered from the literature review defined the parameters for the skin [14], fat [15-17], muscle [15,17-19] layers, and the stoma [17].

A literature review was conducted to identify the typical thicknesses of the abdominal layers (Figure 3), material properties (Figure 4), stoma surgical procedure, and the stoma pouching system application process. The geometries selected for the abdomen layers are illustrated in Figure 3. A controlled and neutral flat abdomen was selected as the best environment for this stage of the investigation as it allowed direct and controlled comparisons of the baseplate range. A more realistic or detailed abdomen may bias results according to the suitability of each product to the specific abdomen example being used. 

The material properties applied to each region of the model are illustrated in Figure 4. The literature review identified a wide range of linear elastic material properties that could be applied to represent the abdominal layers; the tissue stiffnesses for this study were selected from the lower end (lower Young’s modulus) as tissues exhibit softer behaviours under more quasi-static loading (i.e., during the application of an ostomy base plate). The material properties applied to the baseplates were taken from data supplied by Convatec Ltd. and the hydrocolloid adhesive and lamination film layers were simplified to a single part, with an appropriate composite stiffness applied. The key input parameters for the simulation model are summarised in Table 1. 

**Table 1:** Key simulation parameters CAD: computer-aided design; EVA: ethylene-vinyl acetate.

Category	Selection for simulation
Software	Abaqus 2021
Material models	Skin layer: E = 0.5 MPa, ρ = 1000 kg/m^3^ [14]
	Fat layer: E = 0.1 MPa, ρ = 900 kg/m^3^ [15-17]
	Stoma: E = 0.2 MPa, ρ = 1100 kg/m^3^ [17]
	Muscle: E = 0.5 MPa, ρ = 1100 kg/m^3^ [15,17-19]
	Baseplate injection moulding: E=65 MPa, ρ = 900 kg/m^3^
	Baseplate hydrocolloid layer: E=3.1 MPa, ρ = 900 kg/m^3^
Study type(s)	3D dynamic explicit
Dimensions	Based on nominal Convatec CAD data and approximated abdomen geometries
Geometry features	Skin layer and baseplate layers modelled with shell elements
	Fat, muscle, and stoma modelled with solid elements
	3.5 mm depth baseplate EVA thickness: 1.4 mm at region C, 0.8 mm at region D (Figure 2)
	7 mm depth baseplate EVA thickness: 3.0 mm at region C, 0.8 mm at region D (Figure 2)
	Hydrocolloid layer 1.05 mm thick, combined with lamination film for all base plates
Other	Plate adheres to the skin on contact and is assumed to remain joined for the rest of simulation
	Stoma is tied to skin surface and fat layer
	Sliding is allowed between the fat and muscle layers
	Normal motion prevented at the base of the fat layer

For ostomies, maturation (eversion) of the stoma requires suturing through the skin layer, the very top of the fat layer and through the intestinal wall which is pulled into contact with the skin layer [20]. This information was used in the construction of the simulation model to theoretically join the stoma to the skin layer. Further information on the extent of the bonding between the outside of the intestinal wall and the fat and muscle layers was not readily available.

Each baseplate product had a central hole cut to 26 mm, providing a minimum 1 mm border between the stoma and hydrocolloid layer. Each plate was applied to the abdomen in a three-step process: (1) a pressure resulting in 100 N of total force is gradually applied to the inner convex face of the EVA moulding on the baseplate. This aimed to approximate the initial location and application of the baseplate; (2) the force from step 1 is maintained and another 100 N force is gradually applied, distributed across the whole hydrocolloid layer, which aimed to approximate the pushing and smoothing steps; (3) all forces are released, allowing the assembly to relax and settle into the shape experienced when at rest with the baseplate adhered to the abdomen.

Output Parameters

**Figure 5:**
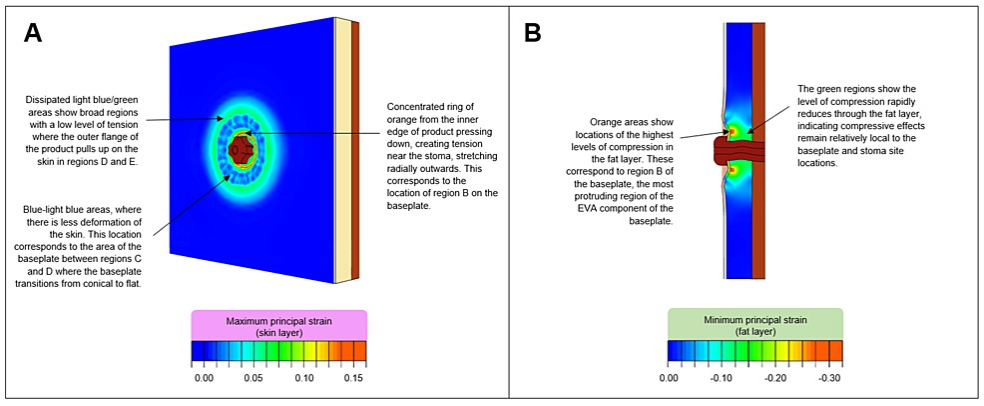
Annotated examples of (A) maximum principal strain response in the skin and (B) minimum principal strain response in the fat

Two sets of outputs, maximum principal strain (MaxPS) and minimum principal strain (MinPS), were generated based on key terminology identified in the literature pertaining to convexity in stoma care (Figure 5). MaxPS has been used to measure the outwards effect of the baseplate on the abdomen skin layer. Elements in the skin layer of the FE model are coloured according to the level of greatest tension or stretch experienced across each one. MinPS has been used to measure the downwards effect of the convex baseplate on the abdomen fat layer. Elements are coloured according to the peak level of compression (or squeezing).

Results

Comparative Outputs

**Figure 6:**
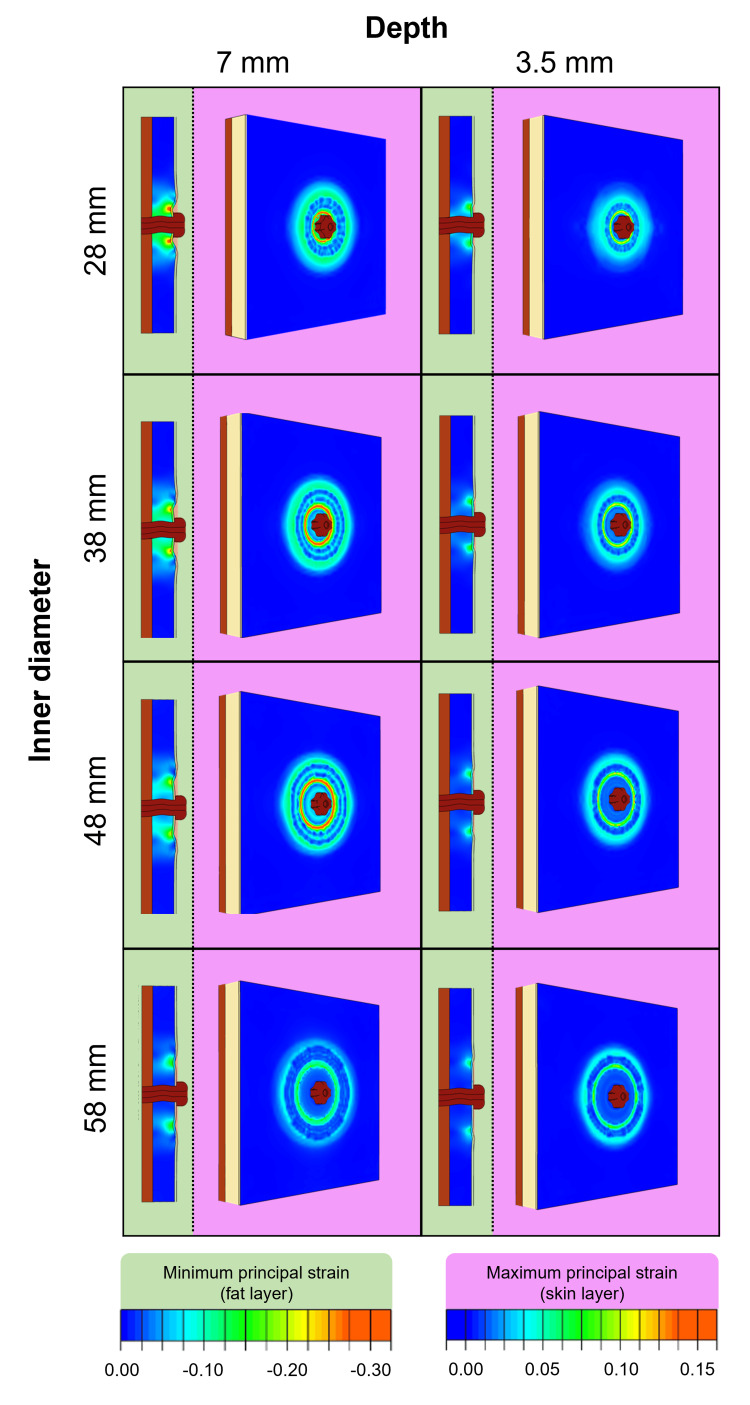
Comparative outputs for baseplate range after application to flat abdomen model Maximum principal strain on the skin (pink columns) and minimal principal strain in the fat (green columns). ID: internal diameter.

Strain outputs for the baseplate range are shown in Figure 6, where MaxPS and MinPS represent skin tension and fat compression produced through convexity, respectively. For all baseplates, the magnitudes of the MaxPS and MinPS in the abdomen was found to depend on both the inner diameter and depth of convexity for each product. The greatest skin tension (MaxPS) and fat layer compression (MinPS) were found to be in the region directly under the inner diameter of the EVA baseplate (region B). These results show that the regions where greatest skin tension and fat layer compression occur vary based on the inner diameter of the EVA component for each product (region B). For all baseplates, the skin had areas of low level tension in the peripheral regions of the baseplates (regions D and E) created by the adhesive on the flange of the product lifting the skin. The 7 mm depth baseplates generated a higher level of central skin tension and fat layer compression than the 3.5 mm depth baseplates.

## Discussion

In this FE analysis, we simulated a range of stoma system baseplates on the abdomen to evaluate the effect of convexity on skin tension and fat compression. The simulations applied the baseplates to the same idealised flat abdomen, allowing for direct comparisons between the full baseplate range and for novel insights into the mechanics of convexity. FE modelling is an important tool that has been used to investigate pressure injuries and their prevention [21-23]. While an FE model has previously been used to visualise the biomechanics of the abdominal wall at different stoma locations [15], our study is the first to apply the method to baseplates around a stoma site.

Our findings show that in addition to generating compression in the fat layer, convex baseplates generate tension in the skin layer under region B. All baseplates produced the greatest skin tension and fat compression at the inner diameter of the EVA component (region B), corresponding to the peristomal skin being drawn outwards and the fat tissue being pushed down. Both the depth and inner diameter of baseplates were shown to affect the location and magnitude of the tension and compression experienced: the deeper baseplates (7 mm depth) produced greater strain than their flatter counterparts (3.5 mm depth) and the smallest diameter baseplates produced higher strains than the wider options. This demonstrates the complexity of the interaction between baseplate and abdomen, which is highly dependent on baseplate parameters. As such, there is value in having a range of convexity geometries.

While this work demonstrates the utility of the FE model in simulating the effect of different baseplate convexities on a peristomal abdomen under identical conditions, its clinical validity will need to be further investigated to explore if the simulated loadings and strains correlate with real-world measurements. For example, further refinement of the application process is warranted to better simulate realistic application pressures and sequences. Furthermore, future work could explore the effects of baseplate convexity on approximated real-world geometries instead of the idealised flat abdomen, compare convex products to nonconvex baseplates and between different manufacturers, as well as compare with moldable products.

## Conclusions

The findings of this FE analysis illustrate the role of convexity in ostomy care and the value of having a range of baseplate geometries to address patient-to-patient variation in stoma type and peristomal skin. The convex product range under investigation produced skin tension and fat compression at different locations and magnitudes based on the depth and diameter of convexity. The FE method enables manufacturers and researchers to objectively compare stoma baseplate products and helps address the need for consistent reporting of product characteristics. Future work to explore the clinical utility of these findings is warranted.
